# The SwissSimilarity 2021 Web Tool: Novel Chemical Libraries and Additional Methods for an Enhanced Ligand-Based Virtual Screening Experience

**DOI:** 10.3390/ijms23020811

**Published:** 2022-01-12

**Authors:** Maiia E. Bragina, Antoine Daina, Marta A. S. Perez, Olivier Michielin, Vincent Zoete

**Affiliations:** 1 Molecular Modeling Group, SIB Swiss Institute of Bioinformatics, University of Lausanne, Quartier UNIL-Sorge, Bâtiment Amphipole, CH-1015 Lausanne, Switzerland; maiia.bragina@unil.ch (M.E.B.); antoine.daina@sib.swiss (A.D.); Marta.Perez@sib.swiss (M.A.S.P.); 2 Department of Oncology UNIL-CHUV, Ludwig Institute for Cancer Research, University of Lausanne, Route de la Corniche 9A, CH-1066 Epalinges, Switzerland; 3 Precision Oncology Center, Department of Oncology, University Hospital of Lausanne, CH-1011 Lausanne, Switzerland

**Keywords:** similarity search, ligand-based virtual screening, molecular fingerprints, drug discovery

## Abstract

Hit finding, scaffold hopping, and structure–activity relationship studies are important tasks in rational drug discovery. Implementation of these tasks strongly depends on the availability of compounds similar to a known bioactive molecule. SwissSimilarity is a web tool for low-to-high-throughput virtual screening of multiple chemical libraries to find molecules similar to a compound of interest. According to the similarity principle, the output list of molecules generated by SwissSimilarity is expected to be enriched in compounds that are likely to share common protein targets with the query molecule and that can, therefore, be acquired and tested experimentally in priority. Compound libraries available for screening using SwissSimilarity include approved drugs, clinical candidates, known bioactive molecules, commercially available and synthetically accessible compounds. The first version of SwissSimilarity launched in 2015 made use of various 2D and 3D molecular descriptors, including path-based FP2 fingerprints and ElectroShape vectors. However, during the last few years, new fingerprinting methods for molecular description have been developed or have become popular. Here we would like to announce the launch of the new version of the SwissSimilarity web tool, which features additional 2D and 3D methods for estimation of molecular similarity: extended-connectivity, MinHash, 2D pharmacophore, extended reduced graph, and extended 3D fingerprints. Moreover, it is now possible to screen for molecular structures having the same scaffold as the query compound. Additionally, all compound libraries available for screening in SwissSimilarity have been updated, and several new ones have been added to the list. Finally, the interface of the website has been comprehensively rebuilt to provide a better user experience. The new version of SwissSimilarity is freely available starting from December 2021.

## 1. Introduction

Drug discovery is unarguably a very complex, expensive, labor-intensive, and time-consuming endeavor. Therefore, considerable effort has been undertaken to facilitate and speed up the underlying processes. Notably, scientific progress in the field of protein structure elucidation as well as the development of computational methodologies stimulated the advent of rational drug discovery and virtual screening. The latter is an in silico technique to search within large chemical libraries for compounds that are likely to be active on a particular target. Virtual screening can be performed using either structural information about a target of interest (structure-based virtual screening) or about its known ligands (ligand-based virtual screening). Structure-based virtual screening employs experimental or modeled protein 3D structures to search for compounds that have shape and other properties complementary to protein binding sites. On the other hand, ligand-based virtual screening searches for small molecules that are similar to the known active ligands, exploiting the similarity principle, which claims that similar compounds are prone to have similar bioactivities.

SwissSimilarity is an online tool (http://www.swisssimilarity.ch) that provides a user-friendly interface for ligand-based virtual screening of chemical libraries, to find compounds that are similar to a query molecule. SwissSimilarity offers several compound libraries for screening, including approved drugs, known bioactive molecules, commercially available and synthetically accessible compounds. To calculate molecular similarity, SwissSimilarity proposes various 2D and 3D molecular fingerprints, which encode compounds in different digital formats, useful to quantify chemical similarity [1]. In view of the similarity principle [2], the list of the output compounds from SwissSimilarity is expected to be enriched in molecules that exhibit the same bioactivity as the query compound. Thus, the tool can be primarily used to identify novel potentially bioactive compounds (hit finding), similar compounds with yet chemically different core structures (scaffold hopping) or to find easily accessible compounds for initial structure-activity relationship studies. In addition, SwissSimilarity can be useful to search for similar compounds from marketed drugs or clinical drug candidates (e.g., to support drug repurposing), or among those whose experimental structures have been determined in complex with their protein targets (e.g., to support molecular docking studies). Last but not least, SwissSimilarity can be used to confirm the chemical novelty of de novo designed molecules.

SwissSimilarity was launched in 2015 [3], and since then, it has performed ~150,000 screenings for almost 50,000 unique users (as of 30 November 2021) from all over the world. Interestingly, one-third of those searches occurred in 2021. SwissSimilarity has been used in a number of different drug discovery campaigns [4,5,6,7,8]. As an example, two novel inhibitors of RON receptor tyrosine kinase have been identified with the help of SwissSimilarity and confirmed using in vitro assays [9]. In addition, our web tool was also used to find possible drug repurposing options to treat Alzheimer’s disease [10] and SARS-CoV-2 infection [11]. As another useful application of SwissSimilarity, the website was employed to search for compounds similar to a novel inhibitor of PKMYT1 kinase from the library of crystallized molecules in order to compare the predicted binding mode of the discovered compound with those available for similar molecules crystallized in complex with kinases [12]. 

The first version of SwissSimilarity relied on the use of path-based FP2 fingerprint [13] as a 2D screening approach, as well as several 3D methods such as Electropshape-5D (ES5D) [14], Spectrophores [15], Shape-IT [16], and Align-IT [16]. Since then, new fingerprinting methods for molecular structure description were developed or have become popular. In particular, the extended-connectivity fingerprint (ECFP), introduced in 2010, is currently one of the most popular 2D fingerprints used for similarity search [17]. The ECFP method is based on “splitting” the chemical structure into separate substructures defined by circular atom neighborhoods. Those substructures are assigned to numerical identifiers, which are then projected onto a binary vector of the desired length with the help of a hashing function. ECFP is able to encode a given chemical structure with a high degree of detail, and it demonstrated excellent performance in benchmarking studies [18]. Additionally, the principle behind the ECFP fingerprint was further adapted for 3D similarity calculations via the creation of the extended 3D fingerprint (E3FP) that can encode molecular conformations [19]. Another powerful method, the MinHash fingerprint (MHFP), has been developed by combining the circular principle of ECFP for substructure definition with the w-shingling technique and MinHash hashing scheme, which are used in natural language processing and text mining [20]. Two other molecular descriptors that deserve attention include 2D pharmacophore fingerprints [21,22] and extended reduced graph fingerprints (ErGs) [23]. Both methods describe a molecular structure by defining its pharmacophoric points and the topological distance between them. If a given combination of pharmacophores and distances is present in the molecule, a corresponding bit in a vector is incremented. While ErG fingerprint defines combinations of two pharmacophoric points and one distance, 2D pharmacophore defines combinations of up to three points and three distances, thus describing the molecular structure in more detail. On the other hand, the ErG fingerprint implements fuzzy incrementation, which favors retrieval of actives with different core structures (scaffold hopping). 

Here, we report the release of an updated version of SwissSimilarity, which makes use of new 2D/3D methods for computing molecular similarity: ECFP, E3FP, MHFP, 2D pharmacophore, and ErG fingerprints. In addition, it is now possible to screen for molecular structures having the same Murcko or generic Murcko scaffold [24,25], in which all atoms are converted to carbons and all bonds to single bonds. Moreover, taking into account the continuous expansion of the chemical space, all chemical libraries available for screening within SwissSimilarity have been updated and several new ones added to the list. Last but not least, the interface of the website was comprehensively rebuilt in order to provide a better user experience.

## 2. Materials and Methods

### 2.1. Chemical Libraries Available in SwissSimilarity

The following compound libraries are available in SwissSimilarity: (1) DrugBank (version 5.1.8, open data) that contains FDA-approved, experimental, investigational and withdrawn drugs, as well as illicit compounds and nutraceuticals [26]; (2) Ligand Expo that collects small molecules appearing in experimental structures deposited within the Protein DataBank [27]; (3) Chemical Entities of Biological Interest (ChEBI) [28]; (4) GPCR-Ligand Association database (GLASS) that contains ligands whose interactions with GPCRs have been experimentally validated [29]; (5) Human Metabolome Database (HMDB, version 5.0) that comprises small molecule metabolites detected in the human body [30]; (6) ChEMBL (version 29) that records bioactivity data of compounds with drug-like properties [31]; (7) drug-like, lead-like compounds and fragment-like compounds (available in stock and purchasable via agent) from the ZINC20 database that contains commercially available molecules and other molecules of interest for drug discovery [32]; (8) in-stock, as well as synthetically tangible, catalogs offered by commercial vendors—namely, Enamine, ChemBridge, Maybridge, Asinex, AsisChem, Otava, SPECS, TimTec, Vitas, Life Chemicals, ChemDiv, Innovapharm. In addition, several focused libraries were created from ChEMBL29 data: approved drugs (maximum phase of development 4); clinical candidates (maximum phase of development 1, 2, or 3); active compounds (activity lower than 10 µM, measured in a binding assay with the highest confidence score of 9); GPCR-targeting molecules; kinase-targeting and protease-targeting molecules (activity lower than 10 µM for a target protein belonging to a given target class and measured in a binding assay with a confidence score of at least 7). All the above-mentioned libraries were retrieved from the corresponding official websites as of October–November 2021. 

### 2.2. Preparation of Small Molecules for Fingerprints Generation

All compound libraries were prepared using OpenBabel (version 3.1.1) [13], Filter-it (Silicos-IT [16], version 1.0.2, Wijnegem, Belgium) and JChem Microservices (ChemAxon, version 21.3, Budapest, Hungary, www.chemaxon.com, accessed on 19 December 2021). OpenBabel was used to convert molecules from SDF to SMILES format when needed and to remove duplicates. Filter-it was employed to remove molecules that have less than six heavy atoms or molecular weight more than 1500 g/mol and to keep compounds that contain only H, C, N, O, S, P, B, F, Cl, Br, and I in their largest fragment. Afterwards, the molecules were standardized (dearomatized, neutralized, and dehydrogenized), and their most frequent tautomeric state was generated using JChem Microservices. For 3D fingerprinting, the major protonation state at pH 7.4 was calculated by JChem Microservices and further submitted for conformers generation, which was achieved by RDkit (version 2021.03.4, www.rdkit.org, accessed on 19 December 2021) for E3FP fingerprints (top three low energy conformers, as recommended in the original article [19]) and by JChem Microservices for ES5D vectors (20 conformations in total, for the sake of consistency with the previous SwissSimilarity version [3]).

### 2.3. Fingerprints Generation and Similarity Calculations

FP2 and ECFP with diameter 4 (ECFP4) were generated using OpenBabel (version 3.1.1) in the form of 2048-bit vectors. To enable screening of molecular structures having the same scaffold, Murcko [25] and generic Murcko [24] scaffolds were extracted for each library compound in the form of SMILES using RDKit. More specifically, the generic Murcko scaffold means a simplified scaffold, in which all heavy atoms are substituted by carbon atoms, and bonds are converted to single ones [21,24]. SMILES of obtained scaffold structures were further employed to generate ECFP4 fingerprints. Then, 2D pharmacophore (feature definitions from Gobbi and Poppinger [22]) and ErG fingerprints were obtained with RDKit. MHFP6 (up to 6 bonds), and E3FP fingerprints were calculated using the corresponding packages available through their respective open-source repositories (https://github.com/reymond-group/mhfp for MHFP6, version 1.9.2 and https://github.com/keiserlab/e3fp for E3FP, version 1.2.3, both repositories accessed on 19 July 2021). For E3FP generation, optimized parameters for fingerprinting were chosen [19]. ES5D vectors were generated with the help of OpenBabel by implementing a previously published algorithm [14]. In order to estimate the similarity between a couple of molecules, a Manhattan distance-based similarity score was used for ES5D vectors [33], while the Tanimoto coefficient (i.e., the Jaccard index) was used for FP2, ECFP4, 2D-pharmacophore, ErG, MHFP6, and E3FP fingerprints. In the case of ErG fingerprints, the Tanimoto coefficient in its algebraic form was implemented [23]. 

To better understand and compare the meaning of the similarity values determined by different methods, we calculated the probability that two bioactive small molecules share a common protein target as a function of their similarity calculated using different molecular fingerprints/vectors. The dataset for calculating these probability curves consisted of 10 million pairs of compounds experimentally active on the same target (among 2261 proteins) and 100 million pairs of randomly selected compounds (presumably without a common target). Compounds and their interactions were retrieved from the ChEMBL25 database using the protocol described elsewhere [34].

### 2.4. Design of the Website

The backend of the new SwissSimilarity website was written in Python 3.9, while its frontend was built using HTML5, PHP 7.4.3, and JavaScript. A queuing system, based on Slurm (version 19.05.5), was set up on the calculation server. This allows better control of the system and it increases the user’s comfort with possibilities to monitor the progress or to stop the computation. Users can provide a query molecule directly as SMILES or through the MarvinJS chemical editor (ChemAxon, version 21.2.0, Budapest, Hungary, www.chemaxon.com, accessed on 19 December 2021); the attached MarvinJS Webservices further convert sketched molecular structures into SMILES. ChemAxon JChem Microservices (version 21.3) was also used to generate images of queries and similar molecules from their SMILES for the output page. The input query compound was prepared the same way as the screening libraries with the only exception that the most frequent tautomeric state was not generated for the query structure, to respect the choice of a user.

## 3. Results and Discussion

### 3.1. Compound Libraries Available for Screening

Four main classes of compounds are available within SwissSimilarity: (1) “Drugs” including approved, experimental and withdrawn drugs, as well as drug candidates that reached clinical trials; (2) “Bioactive” compounds including the LigandExpo collection containing small molecules that appear in the structure entries of the Protein Data Bank, the Chemical Entities of Biological Interest (ChEBI) collection, all compounds from the ChEMBL29 database, the GPCR–Ligand Association (GLASS) database, and the Human Metabolome Database; (3) “Commercial” compounds from vendor catalogs and from different ZINC subsets; (4) “Synthesizable” compounds that can be readily synthesized and provided by a shortlist of vendors. In particular, on top of ZINC (drug-like, lead-like, and fragments), it is possible to screen compounds in the following commercially available chemical libraries: Enamine, ChemBridge, Maybridge, Asinex, AsisChem, Otava, SPECS, TimTec, Vitas, Life Chemicals, ChemDiv, and Innovapharm. Additionally, it is possible to perform searches in focused libraries containing biologically active compounds as well as GPCRs-, kinases- and proteases-targeting molecules. 

### 3.2. Methods Available for Screening

Several 2D and 3D methods are available to perform similarity searches using SwissSimilarity. Among 2D methods, users can opt for FP2 [13], ECFP4 [17], MHFP6 [20], 2D pharmacophore [21,22], and ErG [23] fingerprints. All these approaches have already been assessed by their authors in the respective publications. In addition, it is possible to search for compounds that have the same Murcko scaffold or generic Murcko scaffold (in which all atoms are converted to carbons and all bonds to single bonds) as a query molecule. At the same time, 3D-similar molecules can be searched by using ES5D and E3FP. As recommended in the original article, E3FP fingerprints are calculated from the top three low-energy molecular conformations [19], while ES5D vectors are calculated for up to 20 conformers generated for a given molecule, as described previously [3]. For 3D searches, the similarities of all geometries of a query molecule are evaluated against all geometries of each compound in the screened library. Due to the significant computational time required for 3D screening, those methods are not available for very large libraries. Last but not least, SwissSimilarity also features a “combined” 2D/3D screening method, which is based on both FP2 and ES5D similarity metrics and which has been initially implemented in the reverse screening algorithm of the SwissTargetPrediction web tool [35]. More precisely, the returned combined score corresponds to the probability of sharing a common protein target by two bioactive compounds. It is calculated using logistic regression based on two features, i.e., FP2 and ES5D similarity values. This option is also not available for very large chemical collections. 

### 3.3. Estimation of the Similarity Thresholds

**Figure 1:**
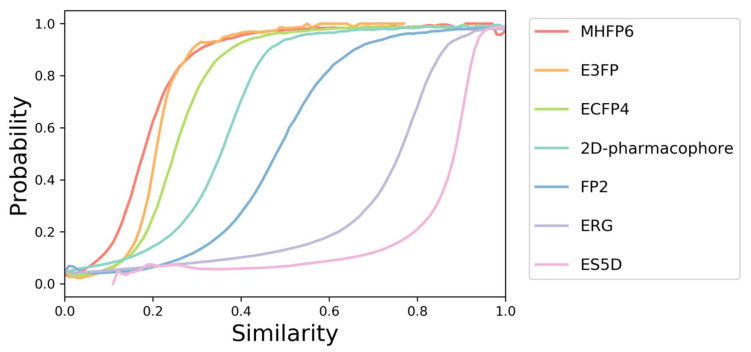
Probability curves. Probabilities to have a common protein target for a pair of compounds with a given similarity calculated with seven different methods (MHFP6, E3FP, ECFP4, 2D-pharmacophore, FP2, ERG, and ES5D). The probability curves were generated using a dataset containing 10 million pairs of compounds sharing the same protein target as confirmed experimentally with binding or functional assays, and 100 million pairs of randomly selected compounds.

Although all similarity scores used in SwissSimilarity vary from 0, for totally dissimilar molecules, to 1, for identical compounds, they cannot be straightforwardly compared. Indeed, similarity values between a given couple of molecules can vary significantly depending on the method chosen for the similarity estimation. For instance, losartan and olmesartan, both Angiotensin receptor type I blockers, have Tanimoto coefficients of 0.72, 0.55, and 0.30 according to their FP2, ECFP4, and E3FP fingerprints, respectively. As a consequence, different similarity thresholds must be applied to these different methods to retrieve similar compounds. In order to define these thresholds, the probability that several molecules share the same protein target as a function of a given similarity value has been calculated for every method available in SwissSimilarity (Figure 1). The probability of ~50% to share the same target was chosen as a lower limit, and thus, SwissSimilarity outputs compounds whose similarities to the query molecule are not less than 0.18 for MHFP6; 0.2 for E3FP; 0.25 for ECFP4; 0.35 for 2D pharmacophore fingerprint; 0.48 for FP2; 0.75 for ERG; 0.83 for ES5D (Figure 1). These curves are provided on the SwissSimilarity website in the FAQ section to help users to estimate the relevance of the retrieved compounds based on the calculated similarity scores. Notably, no similarity score is returned for any of the scaffold-based methods since these screenings output only molecules with scaffolds identical to the query’s one (i.e., all output molecules would have a score of 1.000).

### 3.4. Computation Time

Depending on the number of compounds in a chemical library, on the chosen screening method, and, to a lower extent, on the size and flexibility of the query molecule, the time required to perform the similarity search can vary greatly, from a second to ~20 min. Out of all available screening methods, FP2-, ECFP4-, and (generic) scaffold-based similarity searches provide the best performance in terms of speed, and they can be used to screen all available libraries in SwissSimilarity. In particular, screening of the largest library of 30 million tangible compounds provided by Enamine takes around 200 s with those methods. A queuing system was implemented in order to allow several submitted jobs to run in parallel. An approximate estimation of the computation time is provided on the website for each library/screening method combination to help users to choose the most appropriate options.

### 3.5. Website Usage

**Figure 2:**
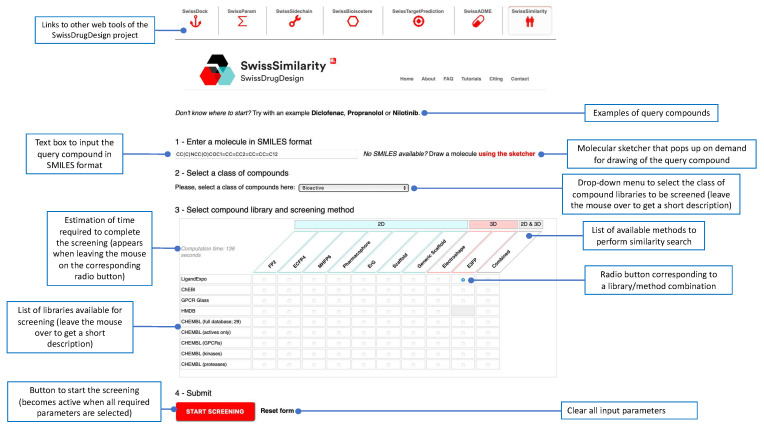
Submission page to set up, parameterize, and launch virtual screening.

**Figure 3:**
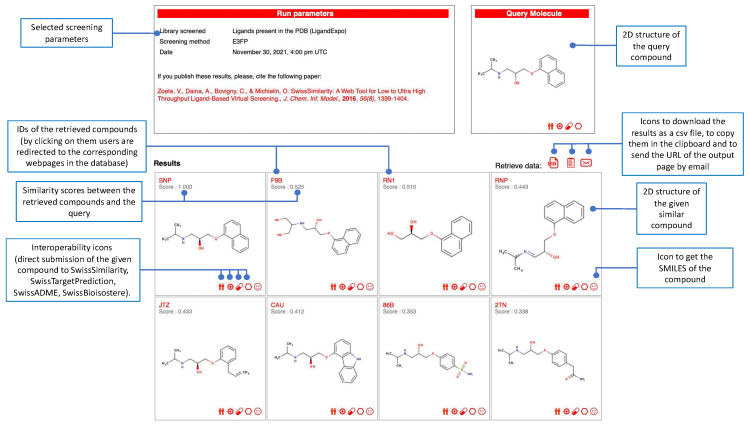
Example of an output page to analyze the most similar molecules to the query by, e.g., accessing the database of origin, or submitting a given compound to other SwissDrugDesign tools through the interoperability icons.

SwissSimilarity is freely accessible and no registration/log-in is required to use the website, which is optimized for recent versions of Google Chrome (www.google.com/chrome/, accessed on 19 December 2021) and Mozilla Firefox browsers (www.mozilla.org/firefox/, accessed on 19 December 2021). Outputs are provided under the CC-BY 4.0 license. To perform virtual screening using SwissSimilarity, users need to (1) provide a query molecule, (2) select a preferred class of compound libraries (Drugs/clinical candidates, Bioactives, Commercially available or Synthesizable compounds), and (3) choose a method and a library, in which the similarity search will take place (Figure 2). By default, a query molecule is submitted in SMILES format in the dedicated text box or, alternatively, it can be drawn in the MarvinJS molecular sketcher, which is displayed on demand. The SMILES box and the sketcher are synchronized, allowing users to visualize any modification of the inputted chemical structure. An estimation of the time needed to run the screening (excluding waiting time in the queue) appears in the upper-left corner of the table when the user moves the cursor from one method/library combination to another (Figure 2). After providing the query compound and clicking on the radio button corresponding to the selected library/method combination, users can launch the calculations by pressing the “START SCREENING” button (Figure 2). The position of the job in the queue and a progress bar are displayed upon job submission and after the initiation of the calculations, respectively. When the screening is completed, an output page opens with retrieved molecules and corresponding similarity values (Figure 3). As already mentioned, the Tanimoto coefficient is used to estimate the similarity between FP2, ECFP4, 2D-pharmacophore, ErG, MHFP6, and E3FP fingerprints, while a Manhattan distance-based similarity score is used for ES5D vectors. Thus, the similarity value should equal 1 for identical compounds and approach zero for totally different molecular pairs. Important to note is that 3D-based screening may in some cases retrieve identical compounds with a score lower than 1.0, due to the slight difference in conformers generation for the query structure and library compounds. Most of those cases are observed when positions of double bonds in aromatic rings are not the same in the query structure and its twin from a compound library. Additionally, molecules retrieved from the scaffold and generic-scaffold searches have, by definition, a score of 1.0; this value is not displayed on the output page, for the sake of clarity. By clicking on the so-called interoperability icons, the virtual hit compounds can be further resubmitted for another similarity search (e.g., for screening another collection, or the same collection with another method) or can be sent to other web tools of the SwissDrugDesign project [36], for example, to SwissTargetPrediction [35] for prediction of their possible protein targets, or to SwissADME [37] for estimation of their physicochemical properties, pharmacokinetics, and drug-likeness. Whenever possible, links are provided to direct users to the original web pages of chemical collections (databases or vendor catalogs). Output from the SwissSimilarity website can be saved in the form of a CSV file or copied to the clipboard of the user’s computer. In addition, users can choose to receive or send the URL of the output page by email. This URL remains active for at least 7 days after the screening. Importantly, command-line access to SwissSimilarity is also provided. The documentation relative to this command-line access can be found on the SwissSimilarity website. 

## 4. Conclusions

The updated version of the SwissSimilarity Web tool has been launched to incorporate novel approaches for molecular fingerprinting and provide access to updated chemical libraries. In addition, the SwissSimilarity interface has been greatly improved to achieve an enriched user experience. Virtual screening with SwissSimilarity is very easy: users only need to submit a reference compound and choose a preferred library/method combination. While the similarity principle is one of the main concepts in medicinal chemistry and chemoinformatics, SwissSimilarity is primarily intended for drug discovery scientists to support their hit-finding and scaffold-hopping activities, as well as studies addressing the structure–activity relationship. As part of the increasingly interoperable SwissDrugDesign project, SwissSimilarity further contributes to the development of a free, online environment for computer-aided drug design.
